# Action of Arsenic in Diseases of the Skin

**Published:** 1882-08

**Authors:** 


HOW CAN THE ACTION OF ARSENIC BE EXPLAINED IN DISEASES OF THE SKIN.

In the treatment of certain diseases of the skin, arsenic is employed very often externally, as well as internally, and especially in malignant diseases of the skin, arsenic is applied externally with good success. The action of arsenic applied externally in lichen, psoriasis, lupus and carcinomatous degeneration has been generally considered to be of a caustic character, which seemed to be proved by the change of tissue and the complete destruction of malignant structures. But every other caustic substance destroys the malignant tissue as well as the healthy structure, which arsenic does not. Others have thought that arsenic forms a composition with the albuminous substances of the diseased structure, but this is not the case. Nitrate of silver and other caustics combine with the albuminous substances of the healthy as well as of diseased structures, destroying both equally. The only way to explain the destructive action of arsenic on these tissues lies in its chemical properties. If we expose a Fowler's solution to the air, thus allowing contact of air and organic substances with the fluid, we will find the arsenic acid changed to arsenious acid. There has been produced an oxidation, one atom oxygen being added: $K_3$ to $O_3+O=K_3$ to $O_4$. The atomic form O is not represented in the air, but the molecule O+O. We must therefore conclude that arsenic acid will be changed to arsenious acid if there is a factor which changes the molecular form into the atomic, and this is the organic substance. It is a well-known fact that arsenious acid gives off its oxygen to reducing substances. If there is a third (organic) substance, giving off oxygen, the arsenic acid will take it up and form arsenious acid. Thus, but a small quantity of arsenious acid will oxydize a considerable quantity of organic substance, by giving off the oxygen to these substances and taking it from another substance, which gives off oxygen. The two facts, that arsenic acid will be easily oxydized to arsenious acid, and that arsenious acid will be easily reduced to arsenic acid, will show the action of arsenic on malignant tumors.

Binz showed first that the active protoplasma in tissues has the capacity in a high degree to thus change the arsenic and arsenious acids. The action of the arsenic is understood to be that arsenic acid seizes upon the oxygen of the cell, forming arsenious acid giving off the oxygen to the same cell, and being reduced to arsenic acid.

Through the blood the arsenic acid is brought in contact with the tissues, and it causes by its oxidation and reduction a degeneration of these tissues, like that of fatty degeneration of the liver in arsenic poisoning. That part of protoplasma which plays a *rôle* in excessive physiological processes, and especially that in the glands, is mostly exposed to the action of arsenic. It is a conceded point that wherever the vitality in organs is at the highest point, there arsenic will act most powerfully, and by that the therapeutical action of arsenic is established. Arsenic acid, in form of a paste, brought in contact with tissues affected with lupus, etc., will be oxydized and reduced alternately, destroying the tissue which is excessive in its vitality. Therefore the nodules of lupus, etc., will undergo necrosis, and the skin will appear, as Kaposi says, as if chopped with a puncheon.—*Monatsehr f. Pract. Dermat.*

THE congress of German surgeons was held at Berlin, President Dr. V. Langenbeck in the chair. Prof. Dr. V. Bergmann spoke on the blood-changes in acute infectious diseases. He does not think all the symptoms of these diseases fully explained by the germ theory (parasitic infection). There are some chemical poisons which reproduce the same symptoms as bacilli. The white blood corpuscles are dissolved not only by the bacilli, but also by certain ferments (pepsine and trypsine).

Prof. Dr. V. Braun thinks extirpation of large malignant strumata not expedient, on account of their rapid growth around the trachea and œsophagus. These tumors grow very often into the veins of the throat, and render an extirpation very dangerous or impossible. Langenbeck and Gussenbauer say that they agree with the author's opinions. Braun says that ligation of the femoral vein near Poupart's ligament is not so dangerous as ligation of the vein and artery. In rare cases, however, it will happen that circulation is impeded after ligation of the vein alone, through tight closure of the venous valves.

Dr. Schwalbe reports his method of radical cure of hernia. He makes injections of alcohol around the hernia. Ranke and Gussenbauer state the safety of this procedure, it being not dangerous, and effecting a shrivelling of the sac. Dr. Michael reports his results with the modified canula of Frenselenburg. In a case of communication between the trachea and œsophagus, he was able to tamponate the trachea with the modified canula permanently. Sonnenberg speaks of the application of permanent baths in the treatment of surgical cases. These baths have been of perfect success in cases where Listerism could not be applied, as in operations of the vagina, rectum, and the bladder.

